# Investment Case for a Comprehensive Package of Interventions Against Hepatitis B in China: Applied Modeling to Help National Strategy Planning

**DOI:** 10.1093/cid/ciaa134

**Published:** 2020-04-07

**Authors:** Shevanthi Nayagam, Polin Chan, Kun Zhao, Elisa Sicuri, Xiaochun Wang, Jidong Jia, Lai Wei, Nick Walsh, Lance E Rodewald, Guomin Zhang, Wang Ailing, Lan Zhang, Joo H Chang, WeiWei Hou, Yingpeng Qiu, Binyan Sui, Yue Xiao, Hui Zhuang, M R Thursz, Fabio Scano, Daniel Low-Beer, Bernhard Schwartländer, Yu Wang, Timothy B Hallett

**Affiliations:** 1 Section of Hepatology and Gastroenterology, Department of Metabolism, Digestion, and Reproduction, Imperial College London, London, United Kingdom; 2 MRC Centre for Global Infectious Disease Analysis, Department of Infectious Disease Epidemiology, Imperial College London, London,UK; 3 World Health Organization China office, Beijing, China; 4 China National Health Development Research Center, National Health and Family Planning Commission, Beijing, China; 5 Health Economics Group, Department of Infectious Disease Epidemiology, Imperial College London, London, United Kingdom; 6 ISGlobal, Hospital Clínic, Universitat de Barcelona, Barcelona, Spain; 7 National Center for AIDS Control and Prevention (NCAIDS), China Center for Disease Control and Prevention, Beijing, China; 8 Liver Research Center, Beijing Friendship Hospital, Beijing, China; 9 Peking University People’s Hospital, Peking University Hepatology Institute, Beijing, China; 10 World Health Organization regional office for the Western Pacific, Manila, Philippines; 11 National Immunization Programme, China Center for Disease Control and Prevention, Beijing, China; 12 National Center for Women and Children’s Health, China Center for Disease Control and Prevention, Beijing, China; 13 Department of Microbiology and Infectious Disease Center, Peking University Health Science Center, Beijing, China; 14 World Health Organization, Geneva, Switzerland; 15 China Center for Disease Control and Prevention, Beijing, China

**Keywords:** hepatitis B, antiviral treatment, investment case, China, modeling

## Abstract

**Background:**

In 2016, the first global viral hepatitis elimination targets were endorsed. An estimated one-third of the world’s population of individuals with chronic hepatitis B virus (HBV) infection live in China and liver cancer is the sixth leading cause of mortality, but coverage of first-line antiviral treatment was low. In 2015, China was one of the first countries to initiate a consultative process for a renewed approach to viral hepatitis. We present the investment case for the scale-up of a comprehensive package of HBV interventions.

**Methods:**

A dynamic simulation model of HBV was developed and used to simulate the Chinese HBV epidemic. We evaluated the impact, costs, and return on investment of a comprehensive package of prevention and treatment interventions from a societal perspective, incorporating costs of management of end-stage liver disease and lost productivity costs.

**Results:**

Despite the successes of historical vaccination scale-up since 1992, there will be a projected 60 million people still living with HBV in 2030 and 10 million HBV-related deaths, including 5.7 million HBV-related cancer deaths between 2015 and 2030. This could be reduced by 2.1 million by highly active case-finding and optimal antiviral treatment regimens. The package of interventions is likely to have a positive return on investment to society of US$1.57 per US dollar invested.

**Conclusions:**

Increases in HBV-related deaths for the next few decades pose a major public health threat in China. Active case-finding and access to optimal antiviral treatment are required to mitigate this risk. This investment case approach provides a real-world example of how applied modeling can support national dialog and inform policy planning.

## Teaser

Approximately one-third of the world’s population with chronic Hepatitis B Virus (HBV) live in China. A comprehensive package of interventions against HBV, including case-finding and antiviral treatment, could reduce HBV-related deaths by 2.1 million and is likely to have a positive return on investment to society over 15 years.

**(See the Editorial Commentary by Dore and Cowie on pages 753–4.)**

In 2016, the World Health Assembly endorsed the first global viral hepatitis targets, calling for elimination of viral hepatitis as a public health threat by 2030. Ahead of the official adoption of these global targets, China led the way in initiating an investment case to inform the development of a national strategy for combating viral hepatitis in 2015. It is estimated that one-third of the world’s population of individuals with chronic hepatitis B virus (HBV) infection live in China where liver cancer is the sixth leading cause of mortality [1–3].

China has made substantial efforts to reduce HBV transmission, including early introduction of infant vaccination in 1992, subsequent integration into the Expanded Program on Immunization in 2002 and abolishment of patient copayments for vaccines in 2005, supported through combined domestic and Global Vaccine Alliance funding [4]. Furthermore, coverage of timely birth-dose vaccine administration was strengthened by promoting birthing within healthcare facilities as part of a rural reform policy [5]. China has therefore witnessed a 97% reduction in hepatitis B surface antigen (HBsAg) prevalence in under-5-year-olds between 1992 and 2014 [6].

Despite these significant prevention efforts, the country continues to face a high burden of adult HBV, with nearly 90 million people estimated to have chronic HBV (although burden estimates from different sources are variable) [2, 7–9] from historical transmission. There are nearly 400 000 annual liver cancer deaths [10, 11]. When the World Health Organization (WHO) targets were launched in 2016, treatment uptake was low and most patients with HBV incurred significant out-of-pocket expenses, as there was limited reimbursement for HBV antiviral therapy. This often leads to high, or even catastrophic, health expenditures associated with treatment [12–15].

Recognizing this high burden of disease and the need to strengthen national viral hepatitis policies, a consultative process was initiated in 2015 by the Chinese Center for Disease Control and Prevention (China CDC) and the WHO, in collaboration with key national and international stakeholders. The aim of this study was to make the case for investment for a comprehensive package of interventions against HBV that would deliver substantial health and economic benefits in China. This study was part of the evidence presented as policy briefs to the government. We will discuss lessons learned for investment case methodology for viral hepatitis and how applied modeling can be used to translate policy into practice.

## METHODS

### Overview

The investment case was performed collaboratively between researchers at Imperial College London (United Kingdom), the WHO’s China Office, China CDC, and other key stakeholders in China.

We developed a mathematical model to address the priority policy questions under consideration: to evaluate the impact, costs, and return on investment (ROI) of a comprehensive scale-up package of prevention and treatment interventions. The structure of the epidemiological model was based on a previously published dynamic transmission HBV model and has been described elsewhere [16]. In brief, we used a simulation model of the hepatitis B epidemic, structured by age and sex and fitted to empirical data on HBsAg and hepatitis B e antigen (HBeAg) prevalence in 1992, 2006, and 2014 [6, 17], and liver cancer mortality in 2012, with an HBV-related liver cancer population attributable fraction of 70% [18, 19]. The model incorporates demographic data, coverage of existing interventions [17, 20, 21], and assumptions about the natural history of HBV informed by literature review.

### Intervention Scenarios

The model was used to make projections of the incidence of new chronic infections, prevalence, and deaths due to HBV under different intervention coverage scenarios (including a status quo scenario) (Table 1). The model was used iteratively to agree on final scenarios based on outcomes considered achievable, adapted to the local setting and aligned with broader strategic goals.

**Table 1.** Intervention Scenarios Modeled

Description	Infant Vaccination, %	Birth-dose Vaccination, %	HBIG,^a^ %	Peripartum Antivirals,^a^ %	Treatment Eligibility	New diagnosis, %	Linkage to Care and Retention, %
Status quo	Continues at current baseline levels						
Full prevention	100	100	100	100	n/a	…	…
Full prevention + treat those already in care^b^	100	100	100	100	Local guidelines	0	…
Full prevention + case finding 20% and treat all eligible	100	100	100	100	Local guidelines	20	90, 90
Full prevention + case finding 20% and treat cirrhosis only	100	100	100	100	Cirrhotics only	20	90, 90
Comprehensive package of public health interventions	100	100	100	100	Local guidelines	50	90, 90

Abbreviations: HBIG, hepatitis B immunoglobulin; HBV, hepatitis B virus; n/a, not applicable.

^a^Of those requiring intervention. The percentage of case finding represents the percentage of new cases diagnosed in each scenario.

^b^Treatment of those in care, assumes that 3.8% of all persons with HBV are already in care.

In China, given the high historical coverage of infant and birth-dose vaccination and the use of HBIG, we considered a “Full Prevention” scenario as the scale-up to 100% coverage of infant vaccination and 100% coverage of Prevention of Mother-to-Child Transmission (PMTCT) interventions (to include all available methods), recognizing that the exact method chosen to reach this coverage would need to be driven by further implementation research and country-specific considerations on feasibility and cost-effectiveness.

We also considered 4 treatment scenarios, which were added to the “Full Prevention” scenario (Table 1). Within the existing healthcare structure, there are already patients who have been diagnosed with chronic hepatitis B (CHB) and treated suboptimally. Therefore, a scenario of treating those already in care, without extra active case-finding was modeled (“Treatment for Those Already in Care”). We then considered a scenario where case-finding was increased to 20% and antiviral treatment was given to all eligible patients, as recommended by contemporaneously available international guidelines (“Case Finding 20% & Treat All Eligible”). An additional scenario included the impact of a disease prioritization strategy, where treatment was given only to those with cirrhosis (“Case Finding 20% & Treat Cirrhosis Only”). The final scenario included the full suite of prevention interventions and high and high coverage level of 50% case-finding and treatment of all those eligible for treatment, which we refer to throughout this article as the “Comprehensive Package of Public Health Interventions.”

### Economic Analysis

An overall societal perspective was taken for the analysis, incorporating both intervention and wider nonintervention costs, and co-financing strategies were evaluated. Nonintervention costs include health system and household costs associated with providing care to those with liver disease (also referred to as “care costs”) and the costs of premature death [14]. Costs of premature death were calculated based on the human capital approach [22] and quantified by the net present value of potential future earnings lost from the year of HBV-related death up to 65 years of age. Average annual income was used to represent potential future earning losses, factoring in a 5% national unemployment rate. Costs (presented in 2015 US dollars) were adjusted for inflation [23] and discounted at 3% per annum, in keeping with WHO guidelines [24] (Table 2). For the baseline analysis, the cost of tenofovir used was $290 per person-year (1800 renminbi [RMB]), which corresponded to the price of tenofovir contemporaneously available to the national human immunodeficiency virus (HIV) program in China.

**Table 2.** Cost Parameters

Intervention Costs	*Component*	*Price, USD*	Source and Comments
Infant vaccination	Unit cost (per dose)	0.63	China CDC
	Delivery costs	0.81	China CDC
Birth-dose vaccination	Unit cost (per dose)	0.63	China CDC
	Delivery cost	0.81	Assume same as infant vaccination
HBIG	Unit cost	16.13	CDC China
	Delivery cost	1.61	Assume double cost of birth dose
Peripartum antivirals^a^	Drug (tenofovir)	97	HIV price of tenofovir (4 months)
	Delivery	142	Unpublished data^b^
Getting someone into care	Case finding	200	Assumption, based on HIV case- finding costs
	Initial diagnostic evaluation	85	Unpublished data^b^
Annual monitoring	Yearly monitoring	114	Unpublished data^b^
Antiviral therapy (annual)	Lamivudine	748	Reform 2015
	Tenofovir	290	Price of tenofovir available to HIV program in China (2015) [25]
Nonintervention costs (costs of care)^c,d^	*Component*	*Stage of Disease*	*Cost, USD*
Direct	Direct medical		
	Annual Outpatient Cost	CHB	591^e^
		CC	1128^f^
		DC	1416
		HCC	1251
	Annual Inpatient Cost	CHB	228
		CC	302
		DC	1426
		HCC	4048
	Cost of medicines self-purchased	CHB	188^e^
		CC	134^e^
		DC	251
		HCC	218
	Direct nonmedical	CHB	373
		CC	718
		DC	777
		HCC	567
Indirect	…	CHB	256
		CC	440
		DC	741
		HCC	531
Other economic parameters	*Estimate*	*Source*	
National annual average income	1669 USD	China Statistics Yearbook	
Average age of ending contribution to GDP	65 years	Assumption	
National unemployment rate	5%	World Bank	
Discount rate: costs	3%	WHO guidelines on economic evaluation of cost-effectiveness [25]	
Discount rate: health benefits	3%		
GDP per capita	7505 USD (2014)	National Bureau of Statistics, 2014	
Conversion rate	1 USD = 6.2 RMB	OANDA	
Inflation rate	3%	World Bank	

Costs in this table are presented in USD (1 US$ = 6.2 RMB).

Abbreviations: CC, compensated cirrhosis; CDC, Center for Disease Control and Prevention; CHB, chronic hepatitis B; DC, decompensated cirrhosis; GDP, Gross Domestic Product; HBV, hepatitis B virus; HCC, hepatocellular carcinoma; HIV, human immunodeficiency virus; RMB, renminbi; OPD, outpatient department; USD, US dollars; WHO, World Health Organization.

^a^Assume 4 months of antiviral drug and 6 months of diagnostics.

^b^China National Health Development and Research Center (Chang et al, manuscript under preparation—see Supplementary Appendix).

^c^Source: Hu et al [14]; only Beijing costs included.

^d^Assumed that 15% of patients with HBV will already be “in care.”

^e^Assumed that after the intervention, these were not included.

^f^Assumed that after the treatment intervention, these were halved.

The primary outcome measure was the ROI on the “Comprehensive Package of Public Health Interventions,” which we defined as the ratio between money defrayed in the collateral costs compared with a “status quo” projection to the money invested in the interventions.

Multivariate probabilistic sensitivity analysis was performed using cost parameter ranges of 0.5–2 times the default estimate. Sampling was performed using the Latin Hypercube method.

## RESULTS

### Epidemiological Projections

**Figure 1.**
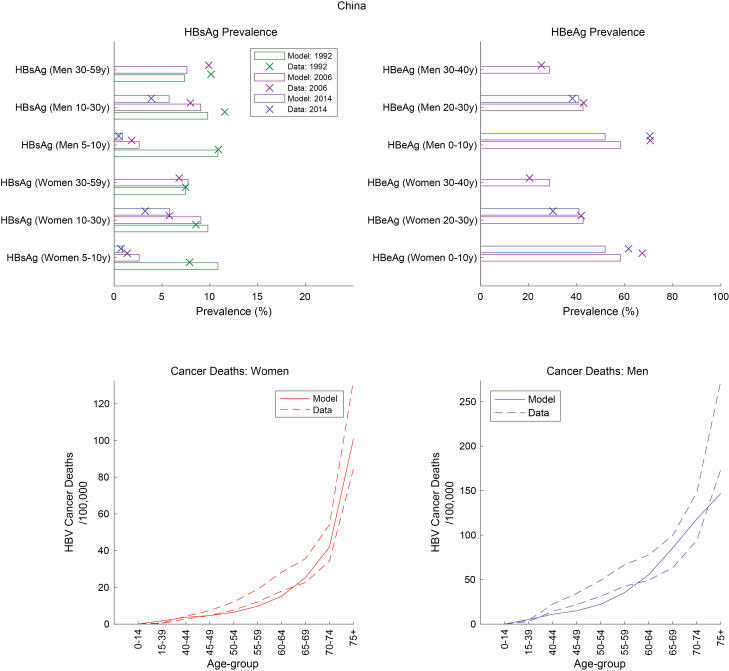
Panels representing the calibration of data against models for HBsAg prevalence, HBeAg prevalence, and cancer deaths. Abbreviations: HBeAg, hepatitis B e antigen; HBsAg, hepatitis B surface antigen; HBV, hepatitis B virus.

The fit between the modeled results and the data for HBsAg prevalence, HBeAg prevalence, and HBV-related cancer deaths can be seen in Figure 1; for the year 2015 this represents a modeled baseline of 81 million persons living with chronic HBV infection, 54 000 new chronic infections, and 570 000 HBV-related deaths.

At status quo levels of intervention coverage, the incidence of new chronic infections would fall from 6500 per year in 2030 to less than 500 per year in 2050 and the number of people living with HBV will decline from 60 million to 32 million over the same period (Supplementary Figure 1). This decline in incidence and prevalence of chronic HBV at status quo levels is seen because coverage of prevention interventions has been strong in China and the cohort of those already infected with CHB are aging.

**Figure 2.**
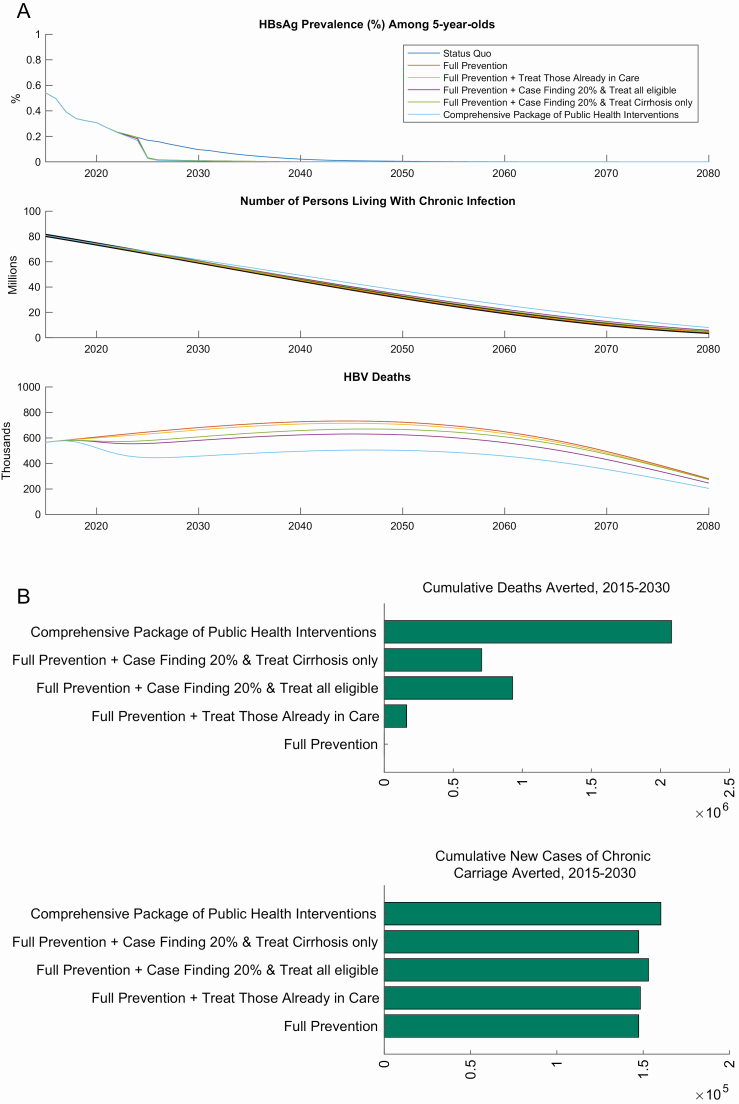
*A*, Impact of intervention scale-up on HBV. Panels representing HBsAg prevalence among 5-year-olds, number of people living with HBV, and HBV-related deaths. *B*, Cumulative 15-year impact on deaths and incidence in China. Abbreviations: HBsAg, hepatitis B surface antigen; HBV, hepatitis B virus.

However, without a change in strategy, there will still be 60 million people living with HBV in 2030 and the major public health threat will come from HBV-related deaths, which will continue to rise and are projected to be 680 000 annually in the year 2030. Without scale-up of treatment and continuing at status quo we project there to be 10 million HBV-related deaths cumulatively over 15 years (2015–2030), including 5.7 million HBV-related cancer deaths (Figure 2).

The scale-up of prevention efforts alone (“Full Prevention”), would avert 150 000 new chronic infections over 15 years compared with the status quo. The main impact of the scale-up of case-finding and treatment strategies would be seen on HBV-related mortality, which would yield the following 15-year HBV-related mortality benefits when compared with the status quo; “Treat Those Already in Care” would avert 162 000 deaths, “Case Finding 20% & Treat All Eligible” would avert 930 000 deaths, “Case Finding 20% & Treat Cirrhosis Only” would avert 706 000 deaths (Figure 2 and Supplementary Table 1).

The “Comprehensive Package of Public Health Interventions” has the highest impact and could avert nearly 160 000 further new chronic infections and 2 million HBV-related deaths (985 000 of which will be cancer deaths) between 2015 and 2030, compared with the status quo.

### Economic Projections

For the comprehensive public health program, the total costs of the intervention components alone would be $2.6 billion (16 billion RMB) in 2020, rising to $3.5 billion (22 billion RMB) in 2030, and would have decreased to $1.8 billion (11 billion RMB) by 2050. Over 15 years, 97% of the cumulative costs would consist of case-finding and treatment costs.

**Figure 3.**
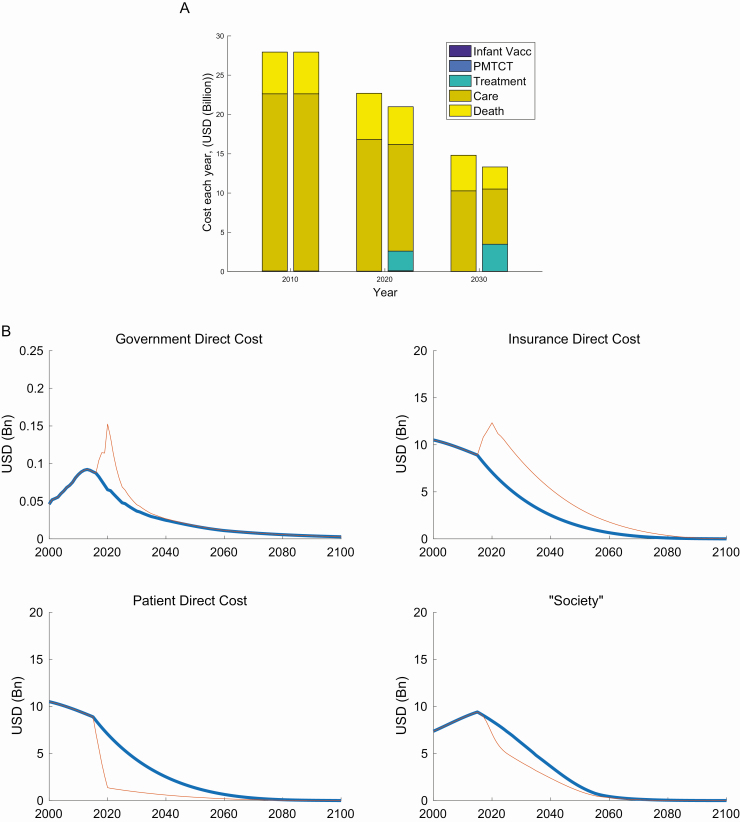
*A*, Comparison of cost projections at status quo versus the comprehensive package. The left bar at each time point represents status quo costs, and the right bar represents the comprehensive package scenario. Costs are presented in USD (1 US$ = 6.2 RMB). *B*, Copayment scenarios for the comprehensive package. Note that the first panel is not to the same scale as subsequent panels. Costs are presented in USD (1 US$ = 6.2 RMB). The blue lines represent the status quo scenario, and red lines represent the comprehensive package of interventions. Abbreviations: Bn, billion; PMTCT, prevention of mother-to-child transmission; RMB, renminbi; USD, US dollars; Vacc, vaccination.

Accounting for the wider nonintervention costs, this combined total is projected to be $23 billion (141 billion RMB) and $15 billion (92 billion RMB) at status quo, but $21 billion (130 billion RMB) and $13 billion (83 billion RMB) for the “Comprehensive Package of Public Health Interventions” in 2020 and 2030, respectively (Figure 3A).

Cumulative costs of premature death are estimated to be $89 billion (549 billion RMB) between 2015 and 2030 at status quo but reduced to $68 billion (423 billion RMB) with the comprehensive package, meaning that nearly $20 billion (126 billion RMB) of productivity losses would be averted. As the new interventions start to take effect, direct medical costs are also displaced as fewer people are in late stages of disease.

Figure 3B shows how costs are partitioned between the different parties over time to fund a comprehensive package under the proposed copayment scenario; government and insurance spending would increase by $0.09 billion (0.5 billion RMB) and $5.2 billion (32 billion RMB), respectively, in 2020 compared with the status quo. Government spending will see the biggest relative increase in its projected spending, but this will be for a short period of time during the active case-finding. Conversely, costs to the patients and society would be $5.7 billion (35 billion RMB) and $1.3 billion (8 billion RMB) less, respectively, than at status quo. This contrasts sharply with the scenario in which patients would continue to pay out-of-pocket at contemporaneous costs for tenofovir for treatment of hepatitis (nearly $3000 per person per year), which would be 160% of average annual incomes, for medication alone.

### Return on Investment From a Societal Perspective

Between 2015 and 2030, investing $42 billion (258 billion RMB) into a comprehensive package of interventions is projected to give an economic return of $65 billion (406 billion RMB) from an overall societal perspective. This would amount to an ROI of $1.57 per US dollar invested and could avert 2.1 million deaths by 2030. These economic returns are largely driven by the reduced costs of premature death (due to increased life expectancy and therefore increase in income generated) and the reduced medical costs of management of end-stage liver disease as less people are in late stages of disease (reduced care costs). Cumulatively, over 15 years, investing in the comprehensive package reduces economic losses due to premature death by 30% and care costs by 23%.

Uncertainty around the cost estimates is represented in Supplementary Figure 2. Although exact quantifications about the magnitude of benefits remain uncertain, the direction of the economic benefit is clear.

**Figure 4.**
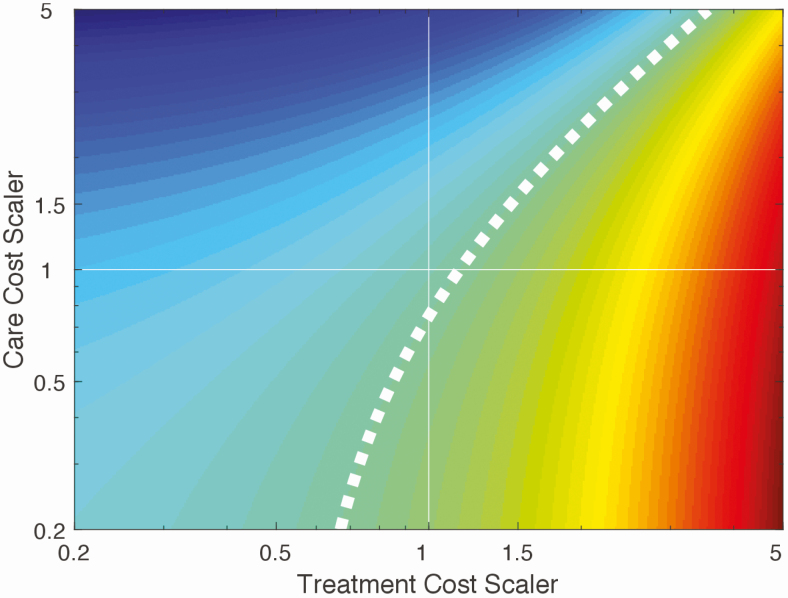
Sensitivity analysis shows the effect of varying treatment costs and care costs on the likelihood that a comprehensive package will be cost saving. The current base-case estimate is represented by the point of intersection of both axes at 1, 1. The dashed white line represents the boundary (“frontier”) between whether the intervention will be cost saving or not. The shading to the left of this frontier (green and blue) indicates combinations of both scalers, which would mean that the intervention would be more cost-saving than the base-case estimate, and to the right (green to red) indicates fewer cost saving combinations.

Figure 4 examines how variation in 2 of the most uncertain cost inputs, namely care costs and treatment costs, would affect whether the comprehensive package would provide a positive ROI. If our study has underestimated treatment costs, the comprehensive package is unlikely to provide a positive ROI.

## DISCUSSION

Maintenance of prevention interventions against HBV infection are essential in China. However, the major public health impact now will come from averting HBV-related deaths. Without a scale-up of HBV testing and treatment, there will be 10 million HBV-related deaths, including 5.7 million HBV-related cancer deaths between 2015 and 2030. However, a comprehensive public health program for HBV with full coverage of infant vaccination, PMTCT, and high active case-finding and treatment with reduced-cost tenofovir covered in a national program could avert 2.1 million deaths by 2030 and is likely to provide a positive ROI from an overall societal perspective (ROI = $1.57 per US dollar invested).

China was one of the first countries to initiate an investment case for viral hepatitis in 2015 and, given its high burden of disease, is uniquely poised to make a significant global impact on the HBV epidemic. An increased effort in China alone could result in a 12% reduction of the total 17 million predicted global HBV-related deaths between 2015 and 2030 [16]. This approach proposes a unique framework for viral hepatitis strategy planning using a real-world case study and highlights how applied modeling can help initiate national dialog and inform policy change. Our study also highlighted how out-of-pocket patient costs will be high if patients had to pay the original price of tenofovir, particularly without reimbursement. Furthermore, achieving lower costs for tenofovir is key to there being a favorable ROI (Figure 4). In May 2016, the government of China successfully negotiated the price reduction of tenofovir to less than one-third of its original price (from 1500 RMB [$237] to 490 RMB [$77] per month) [26, 27]. Concurrently, prices of generic entecavir, which was historically more expensive than tenofovir, fell to about 300 RMB per month. In February 2017, these 2 WHO-recommended first-line anti-HBV medications were included in the national reimbursement drug list, allowing more patients access to the medications [28]; and in November 2017, China announced its national comprehensive action plan for viral hepatitis [28]. At the end of 2018, with patent expiry of tenofovir, the National Insurance Agency negotiated for central pooled procurement supplying 11 provinces (comprising the main treatment burden) as a pilot, reducing the prices for both first-line medications to $30-35 per person-year. Extension of this pilot to the whole country in 2019 reduced prices further to $10 per person-year, effectively removing a major barrier in China to scale-up of treatment, which was the cost of medicines [25].

Previous epidemiological modeling analyses have examined the HBV epidemic in China [29, 30]; however, this study, to our knowledge, is the first analysis using an ROI approach to develop an investment case for a comprehensive package of interventions for HBV. Its strengths include a collaborative process of stakeholder engagement, use of a high-quality epidemiological model calibrated against nationwide seroprevalence data, consideration of the wider societal costs of premature death, as well as evaluating co-financing strategies. In the context of the subsequent adoption of the WHO global targets in 2016, such work has become essential to guide how countries might aim to achieve them.

Although the use of cost-effectiveness analyses (CEAs) predominates the recent health economic literature [31], the lack of clear data and consensus on cost-effectiveness thresholds and the indirect way in which CEAs respond to key questions about affordability and financial impact arguably limit their use when answering questions about budget expansion and value for money that a new investment would bring, especially in the context of low- and middle-income countries (LMICs). This informed our decision to adopt an ROI approach for this study, which has been used in evaluating other public health interventions [32] and in the field of HIV [33, 34]. A review of ROI of public health interventions revealed wide ranges of returns from 1 to 14.3 and methodology was heterogeneous between studies [32].

The human capital method that we used to represent the cost of premature death is based on the theoretical and simplifying assumption that premature death means that the economy of the country forgoes potential future gross domestic product contributions pertaining to that individual. This method has been used previously in other disease areas, including malaria [35] and cancer [36]. However, this approach considers only the market value of health. Other frameworks have been used to encompass broader aspects including the nonmarket value of a healthy life. One of these approaches includes the concept of “value of additional life-years” (VLYs), which has been used, along with Gross Domestic Product growth, in the full-income approach by the Global Health 2035 Lancet Commission in 2013 [37]. They estimated that the VLY to be an average of 2–3 times the per-person income in LMICs. Using such an alternative approach would place higher weight on the value of health interventions and therefore provide an even higher ROI than we have estimated in our study.

Although we have indicated the significant health gains, costs, and economic savings related to a scale-up of a comprehensive package of interventions against HBV, how these targets can be reached and how operationally, financially, or politically feasible this will be remain to be determined. First, there are operational questions about how scale-up of case-finding to increase diagnosis rates and treatment on such a large scale can be achieved, given the complexities of the Chinese healthcare system and provincial variation in implementation. Other than during pregnancy, systematic screening for HBV is not performed in China. Innovative case-finding strategies have been used in other regions: for example, active case-finding for HBV has been shown to be feasible and cost-effective in other high-endemic settings [38].

The economic projections, which have shown that case-finding and treatment costs account for the majority of the overall comprehensive package, are critically dependent on price negotiations for the reduction of tenofovir therapy for HBV monoinfection to the price available in the HIV program, which, at the time of this study, was just 10% of the tenofovir price for HBV treatment. China’s economic position as an upper-middle-income country, especially at a time of reduced donor funding internationally and the lack of any current global hepatitis-funding mechanism, is likely to mean that expansion of a hepatitis program will need to be supported by domestic funding. In the HIV response, China has shown that migration from external to domestic funding has been possible, now funding more than 95% of its HIV response from domestic sources.

China-specific data were used where possible to parameterize the model. However, there are limitations of the available data. Despite the significant increase in the number of local population-based cancer registries in China (from 54 in 2008, to 308 in 2014), many fail to meet quality standards for reporting [11]. We used 2012 cancer estimates reported to the International Agency for Research on Cancer (IARC), which only include data from registries with high reporting standards and therefore may not be representative of the country overall [18]. Improvement in the validity and completeness of cancer registries is essential to provide more accurate predictions of the burden of HBV-related disease. There are also other factors which could affect the projections including the uncertainty in current estimates of the number of persons already diagnosed and in care, the impact of the recent relaxation of the 1-child policy [39] and the regional variation in costs of interventions.

Our study has shown that a comprehensive effort to address chronic HBV and related liver disease in China will help the country move closer to achievement of the recently adopted WHO hepatitis elimination goals and Sustainable Development Goals, provide economic returns on investment to society, and is likely to have a large impact on the global state of the hepatitis B epidemic. This study provides a strong policy framework that other countries can follow to tackle their HBV epidemic.

## Supplementary Data

Supplementary materials are available at *Clinical Infectious Diseases* online. Consisting of data provided by the authors to benefit the reader, the posted materials are not copyedited and are the sole responsibility of the authors, so questions or comments should be addressed to the corresponding author.

ciaa134_suppl_Supplementary_Material

## Notes

***Author contributions.*** All authors were part of the consultative investment case process and contributed to scenario development. S. N., T. B. H., P. C., and D. L.-B. designed the study. S. N. and T. B. H. contributed to the development of the economic component of the model, carried out the analyses, and wrote the manuscript. E. S. contributed to the economic module development. K. Z., J. H. C., W. H., Y. Q., B. S., and Y. X. contributed to the costings. All authors read and approved the final manuscript.

***Acknowledgments.*** The authors acknowledge the support of the UK Medical Research Council (MRC) and the UK Department for International Development (DFID) who jointly funded (under the MRC/DFID Concordat agreement) the clinical research fellowship that supported S. N. during the conduct of this study. The authors acknowledge the support of the UK National Institute for Health Research Biomedical Research Centre at Imperial College London for infrastructure support.

***Financial support.*** The World Health Organization provided funding support of the Hepatitis Investment Case process (to Y. Q., S. N., K. Z., and T. B. H.).

***Potential conflicts of interest.*** T. B. H. reports personal fees from the World Health Organization and grants from the Global Vaccine Alliance/Bill and Melinda Gates Foundation outside the submitted work. S. N. has also received personal fees from WHO outside this submitted work. All other authors report no potential conflicts. All authors have submitted the ICMJE Form for Disclosure of Potential Conflicts of Interest. Conflicts that the editors consider relevant to the content of the manuscript have been disclosed.
