# Supplementary material for: Investment Case for a Comprehensive Package of Interventions Against Hepatitis B in China: Applied Modeling to Help National Strategy Planning
Source: Clin Infect Dis. 2020 Apr 7;72(5):743–52. doi: 10.1093/cid/ciaa134 (PMC7935389; doi:10.1093/cid/ciaa134)
Supplement: ciaa134_suppl_Supplementary_Material [file ciaa134_suppl_supplementary_material.docx]

**Investment Case for a Comprehensive Package of Interventions against Hepatitis B in China; applied modelling to help national strategy planning.**

## S. Nayagam,^1,2^ P. Chan,^3^ K. Zhao,^4^ E. Sicuri,^5,6^ X. Wang,^7^ J. Jia,^8^ L. Wei,^9^ N. Walsh,^10^ L.E. Rodewald,^3^ G. Zhang,^11^ W. Ailing,^12^ L Zhang,^3^J. Chang,^4^ W. Hou,^4^ Y. Qiu,^4^ B. Sui,^4^ Y. Xiao,^4^ H. Zhuang,^14^ M. Thursz,^1^ F. Scano,^3^ D. Low-Beer,^15^ B. Schwartländer,^15^ Y. Wang,^16^ T. B. Hallett.^2^

**SUPPLEMENTARY APPENDIX**

**Contents**

1. Supplementary Methodology
2. Supplementary Results
3. Supplementary Discussion
4. Supplementary Figures
5. **Supplementary Methodology**

Two consultative stake-holder meetings were convened in Beijing, China in 2015 to develop and parameterise the modelled scenarios. The scenarios modelled included considerations around existing coverage of interventions, feasibility and acceptability and presumed capacity of the local health care system to absorb scale-up of interventions. 90% coverage of testing and 80% treatment to target levels, as was subsequently adopted by WHO, was felt not to be a realistic target level by national stake holders for the context of high burden China and was therefore not modelled.

**Intervention Coverage**

National Intervention coverage data was used to parameterise the model. By 2015 intervention coverage had reached 99% for infant vaccination,^1^ 96% for timely birth dose vaccination (which had been high since 2005).^1,2^ Screening for HBsAg with selective HBIG use for those born to all HBsAg mothers has formed part of national recommendations since 2010, and had reached 91% by 2013, although coverage rates nationally are variable^3^. There is currently no systematic population screening for chronic hepatitis B infection in China, therefore there remains uncertainty around numbers tested and aware of their diagnosis.^4^ However, the consultation agreed that about 15% of HBsAg positive persons are aware of their diagnosis and 3.8% are on antiviral therapy, the majority of whom are on sub-optimal regimens with a low barrier to resistance.^5^

**Costings & Economic Model**

A mixed methods approach was used for data inputs; primary data collection, secondary data analysis (published literature, publicly available reports, databases and other government reports), and consensus expert opinion. The latter was used to inform the missing parameters, provide health system context and reflections on scale up.

Intervention costs included costs of infant vaccination, PMTCT (to include birth dose vaccination, HBIG and peripartum antiviral therapy), case-finding, diagnostic work up and monitoring and antiviral therapy. For each component, unit costs and delivery costs were estimated (Table 2). In the absence of primary data on HBV case-finding costs in China, a proxy of HIV case finding costs were used.^6^ For the baseline analysis, the cost of tenofovir used was US$290 (1800 RMB), which corresponded to the price of tenofovir contemporaneously available to the national HIV programme in China. Although entecavir is an equally effective antiviral therapy for CHB, tenofovir was chosen as the primary drug in this analysis due to price and availability at the time of the study.

The costs associated with diagnosis and monitoring of chronic hepatitis B were collected using a microcosting approach. An optimal standardised package of investigations needed for diagnosis and monitoring was established through expert review and based on national guidelines. The unit costs of these investigation were then collected through a multicenter survey of 16 hospitals in China, representing four regions and four levels of hospital (Chang et al, manuscript in preparation).

Direct medical costs are used here to describe costs of management of HBV-related liver disease. This includes outpatient and inpatient costs, as well as costs of self-purchased medications in pharmacies. Direct non-medical costs include transportation costs incurred to reach the place of treatment. These costs were parameterised with Beijing specific costs data taken from published literature.^7^

We used the epidemiological model to output the number of people in each health state over time in each scenario and attached relevant costs to each individual in the model.

Equation 1:

*Total Costs = Intervention Costs + Non-intervention Costs*

Equation 2:

$$\boldsymbol{Investment}_{\boldsymbol{sc}}= {Intervention Costs}_{sc}- {Intervention Costs}_{status quo}$$

$$\boldsymbol{Returns}_{\boldsymbol{sc}}= {NonIntervention Costs}_{status quo}- {NonIntervention Costs}_{sc}$$

$$\boldsymbol{Return on Investment (ROI)}= \frac{\boldsymbol{Returns-Investment}}{\boldsymbol{Investment}}$$

For the co-financing scenarios, the consultation agreed that it should be considered that interventions for prevention of HBV and case-finding would be funded by the government, as part of a public health strategy, which has a precedent in the sectors of Tuberculosis (TB) and HIV case-finding in China. Care and treatment costs, however, are shared between insurance and patient, which are projected under the assumption that the co-payment factor between insurance and patient would increase from status quo levels (equal split between insurance and patient) to 90-10% by 2020 (which reflects the co-payment systems in other disease areas in China which are considered to represent a significant public health threats).

1. **Supplementary Results**

Although historic vaccination scale-up has averted many new chronic infections since its introduction in 1992, given the time-delay between vaccination and impact on HBV-related deaths, the full mortality benefits are yet to be seen. However, an estimated 83,000 HBV-related deaths are likely to have been averted upto 2015, due to historic interventions against HBV, and that number will grow in the coming years (Figure S1).

The comprehensive package will achieve a 20% reduction in HBV-related deaths by 2030 compared to 2015 baseline (Table S1). This scenario shows an initial decline in HBV-related deaths but this is followed by a subsequent slight increase upto 2050 which is likely related to an age cohort effect due to population growth.

**Table S1**: Percentage reduction of HBV-related mortality and new chronic infections by 2030 compared to 2015 baseline. *extra scenario of 80% case finding.

|  | Percentage reduction in HBV-related deaths by 2030 (compared to 2015 levels) | Percentage reduction in new chronic infections by 2030 (compared to 2015 levels) |
| --- | --- | --- |
| Full prevention | - 20 | 98.9 |
| Full prevention + Treat those already in care | - 17 | 98.9 |
| Full Prevention + Case Finding 20% & Treat all eligible | - 2.6 | 99.1 |
| Full Prevention + Case Finding 20% & Treat Cirrhosis only | - 7.5 | 98.9 |
| Comprehensive package of public health interventions | 19 | 99.3 |
| Full Prevention + Case Finding 80% & Treat all eligible* | 40 | 99.6 |

1. Supplementary Discussion

Although a comprehensive package has a significant health impact, it only achieves a 20% reduction in HBV-related mortality by 2030 (compared to 2015 levels), which falls short of the WHO target of a 65% reduction in mortality by 2030. Even if case finding were to be increased to 80%, this would lead to a 40% reduction in HBV-related mortality by 2030. The difficulty in achieving mortality target compared to 2015 baseline, is largely related to the rising rate of HBV related deaths in the decades to come.

Further limitations to our study include the following; Although our study has included projections at different levels of case-finding, we have not specified different methods of case-finding as there is limited data on the bias that these screening methods would have, for example towards specific age groups of disease severity. Finally, although it is increasingly recognised that occult HBV infection has oncogenic properties, we have not analysed this in our study as occult infection would not be picked up through conventional screening programmes which are based on HBsAg testing and robust data on the population prevalence of occult infection and longitudinal data about its natural history are lacking.

1. **Supplementary Figures**

**REFERENCES**

1. WHO/UNICEF Estimates of National Immunization Coverage (WUENIC). Geneva: World Health Organization. <http://apps.who.int/immunization_monitoring/globalsummary/timeseries/tscoveragehepb_bd.html2015>).

2. Liang X, Bi S, Yang W, et al. Epidemiological serosurvey of hepatitis B in China--declining HBV prevalence due to hepatitis B vaccination. *Vaccine* 2009; **27**(47): 6550-7.

3. Cui F, Luo H, Wang F, et al. Evaluation of policies and practices to prevent mother to child transmission of hepatitis B virus in China: results from China GAVI project final evaluation. *Vaccine* 2013; **31 Suppl 9**: J36-42.

4. Lim SG, Amarapurkar DN, Chan HL-Y, et al. Reimbursement policies in the Asia-Pacific for chronic hepatitis B. *Hepatology international* 2015; **9**(1): 43-51.

5. Yu R, Fan R, Hou J. Chronic hepatitis B virus infection: epidemiology, prevention, and treatment in China. *Front Med* 2014; **8**(2): 135-44.

6. Johnson C DS, Baggaley R. Annex 5. Systematic review of HIV testing costs in high and low income settings. WHO/HIV/2015.24, 2015.

7. Hu M, Chen W. Assessment of Total Economic Burden of Chronic Hepatitis B (CHB)-Related Diseases in Beijing and Guangzhou, China. *Value in Health* 2009; **12, Supplement 3**(0): S89-S92.
